# Randomized Trial of Anti-inflammatory Medications and Coronary Endothelial Dysfunction in Patients With Stable Coronary Disease

**DOI:** 10.3389/fcvm.2021.728654

**Published:** 2021-10-15

**Authors:** Allison G. Hays, Michael Schär, Gabriele Bonanno, Shenghan Lai, Joseph Meyer, Yohannes Afework, Angela Steinberg, Samuel Stradley, Gary Gerstenblith, Robert G. Weiss

**Affiliations:** ^1^Division of Cardiology, Department of Medicine, Johns Hopkins University School of Medicine, Baltimore, MD, United States; ^2^Division of Magnetic Resonance Research, Department of Radiology, Johns Hopkins University School of Medicine, Baltimore, MD, United States; ^3^Department of Epidemiology, Johns Hopkins Bloomberg School of Public Health, Baltimore, MD, United States; ^4^Institute of Human Virology, University of Maryland School of Medicine, Baltimore, MD, United States

**Keywords:** inflammation, coronary artery disease, coronary endothelial function, magnetic resonance imaging, flow mediated dilatation

## Abstract

**Aims:** Inflammation plays a critical role in the pathogenesis of coronary artery disease (CAD), however the impact of anti-inflammatory therapies to reduce those processes which promote atherosclerosis in CAD patients is unknown. We aimed to test the hypothesis that anti-inflammatory approaches improve impaired coronary endothelial function (CEF), a driver of coronary atherosclerosis, in stable CAD patients.

**Methods and Results:** We performed a single-center, randomized, placebo-controlled, double-blinded trial to assess whether low dose methotrexate (MTX), low dose colchicine (LDC), and/or their combination (MTX+LDC), improves CEF using non-invasive MRI measures in patients with stable CAD (*N* = 94). The primary endpoint was the MRI-detected change in coronary cross-sectional area from rest to isometric handgrip exercise (IHE), a predominantly nitric oxide-dependent endothelial dependent stressor. Coronary and systemic endothelial endpoints, and serum inflammatory markers, were collected at baseline, 8 and 24 weeks. Anti-inflammatory study drugs were well-tolerated. There were no significant differences in any of the CEF parameters among the four groups (MTX, LDC, MTX+LDC, placebo) at 8 or 24 weeks. Serum markers of inflammation and systemic endothelial function measures were also not significantly different among the groups.

**Conclusion:** This is the first study to examine the effects of the anti-inflammatory approaches using MTX, LDC, and/or the combination in stable CAD patients on CEF, a marker of vascular health and the primary endpoint of the study. Although these anti-inflammatory approaches were relatively well-tolerated, they did not improve coronary endothelial function in patients with stable CAD.

**Clinical Trial Registration:** www.clinicaltrials.gov, identifier: NCT02366091.

## Introduction

Despite advances in contemporary preventive and treatment strategies, coronary atherosclerosis remains prevalent and its manifestations have a high personal and societal toll. Because coronary atherosclerosis is an inflammatory disease (1), there is renewed interest in inflammation as a treatment target (2–4). Endothelial cell injury occurs at the earliest stages of coronary atherosclerosis and inflammatory cells, cytokines, and mediators are involved in all stages of CAD (1, 5). Although coronary atherosclerosis is recognized as an inflammatory process, this important concept is still not applied in the management of patients with, or at risk for, the disease. One important reason is the lack of an established and easily obtained measure of the effect of inflammation on the processes which result in coronary atherosclerosis. Inflammation undoubtedly enhances the development and progression of coronary atherosclerosis via several mechanisms, but endothelial dysfunction is believed to be one common result of these mechanisms (6) and is thus a potential target for medical interventions (6, 7).

One of the principal manifestations of impaired coronary endothelial function is decreased elaboration of nitric oxide (NO) in response to interventions which stimulate endothelial-dependent NO release. NO-mediated changes in coronary cross sectional area and blood flow were historically measured by conventional coronary catheterization-based techniques which are not well-suited to clinical trials in stable patients. Fortunately, non-invasive CEF measures were developed that use 3T magnetic resonance imaging (MRI) to assess endothelial-dependent coronary vasomotor function and these MRI-CEF measures were shown to be reproducible, primarily NO-mediated, and to improve within weeks following LDL lowering with PCSK9 inhibition (8, 9).

Although statins have anti-inflammatory properties (10), cardiovascular event rates remain high in statin treated CAD patients (11), and statins alone do not fully suppress inflammation in many patients (4). Several very recent trials of anti-inflammatory strategies (canakinumab, methotrexate, colchicine) in CAD or myocardial infarction patients reported varied results from reduced events to no benefit (3, 4, 12). It is not clear whether the discrepancies are due to differences in the anti-inflammatory agents or to the populations studied. To date there are no head-to-head comparisons of anti-inflammatory agents in patients with CV disease in a single trial. Likewise, there are no studies of the direct effects of anti-inflammatory strategies on the endothelial processes that contribute directly to coronary atherosclerosis or its progression.

In this study, we performed a randomized double-blinded, placebo-controlled trial to test the hypothesis that anti-inflammatory approaches, namely low dose methotrexate (MTX), low dose colchicine (LDC), and/or their combination, improve impaired local CEF compared to placebo in patients with stable CAD and either elevated markers of inflammation or diabetes/metabolic syndrome, both inflammatory states. We chose these agents because methotrexate and colchicine have been used in clinical practice for decades to treat inflammatory diseases, they are less expensive that canakinumab and, in observational studies are associated with reduced cardiovascular risk (13). The combination of MTX and LDC (MTX+LDC) is used to treat primary biliary sclerosis and its incorporation in one of the study arms is an additional novel aspect of this trial. In addition, to test whether anti-inflammatory strategies have a rapid, direct vascular effect akin to that previously demonstrated by statins (14), we assessed coronary artery endothelial function by MRI and systemic endothelial function by flow mediated dilation (FMD) in the brachial artery after 8 weeks and again after 24 weeks.

## Methods

**Figure 1:**
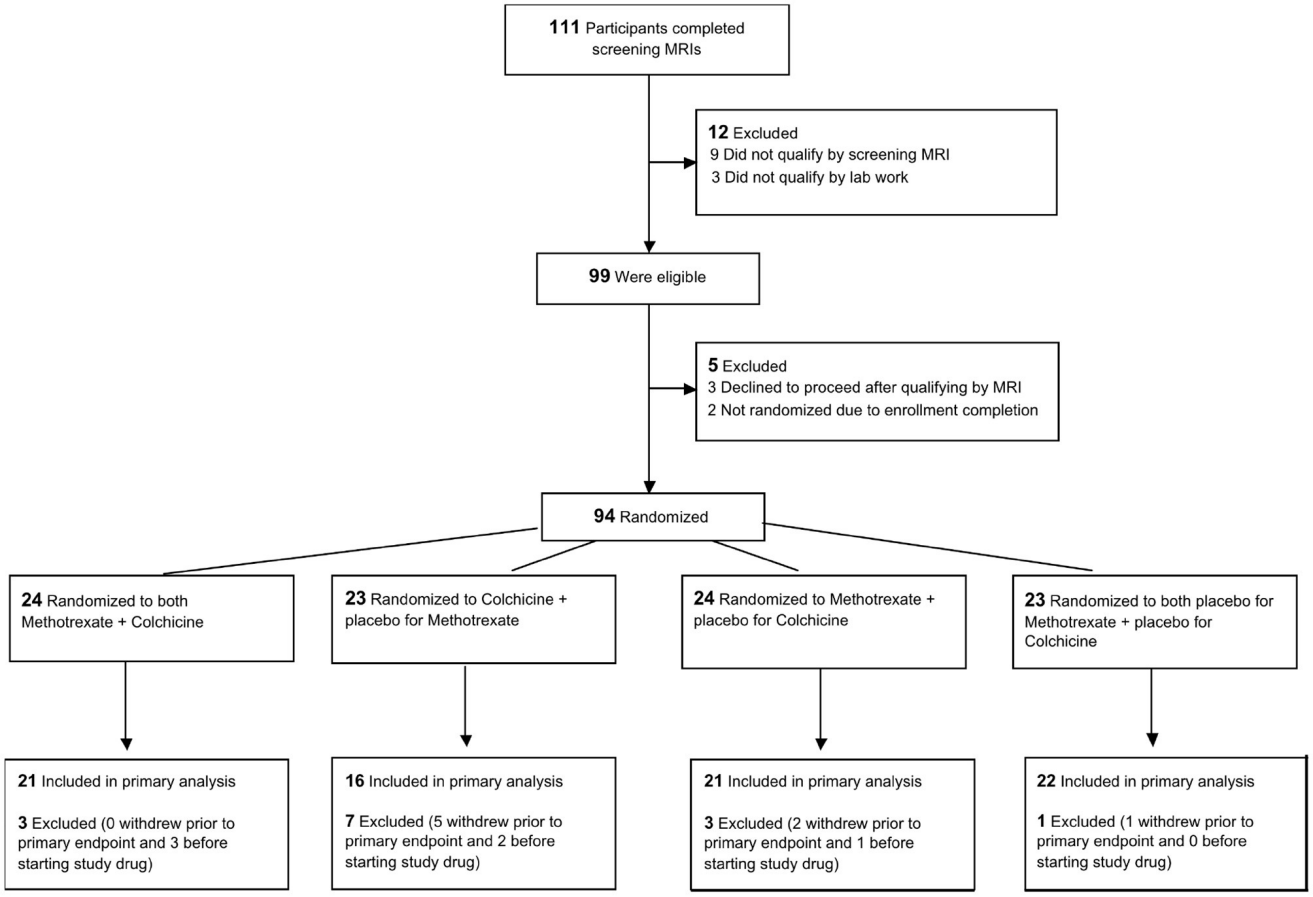
Trial flow chart.

This was a single-center, randomized, double-blinded, placebo-controlled trial with a 2 × 2 factorial design conducted at the Johns Hopkins Hospital and funded by the National Institutes of Health. The purpose was to test the hypothesis that anti-inflammatory strategies improve coronary and systemic arterial endothelial function. Stable CAD patients were recruited from the outpatient clinics at Johns Hopkins Medicine who were on conventional medical therapy (Table 1) and who had either (a) hsCRP >2 mg/L or (b) either the metabolic syndrome or diabetes. Potential participants underwent screening MRI to measure CEF and those with at least one coronary segment qualifying as “abnormal CEF” [defined as no change or a decrease in coronary cross-sectional area (CSA) during isometric handgrip exercise, i.e., a change of ≤0% of the resting value (8, 15, 16)] underwent additional screening procedures (Figure 1).

**Table 1:** Demographic characteristics of trial population.

**Baseline characteristics of trial participants**	**Both MTX and colchicine** **(*n* = 24)**	**Colchicine and placebo for MTX** **(*n* = 23)**	**MTX and placebo for colchicine** **(*n* = 24)**	**Placebo for both MTX and colchicine (*n* = 23)**
Median age (IQR)—year	63.4 (56.9–70.7)	66.8 (56.2–67.9)	61.6 (56.7–68.7)	63.9 (55.9–69.5)
Male sex—no. (%)	21 (87.5)	21 (91.3)	18 (75.0)	21 (91.3)
Caucasian—no. (%)	21 (87.5)	19 (82.6)	20 (83.3)	20 (87.0)
Black—no. (%)	2 (8.3)	3 (13.0)	2 (8.3)	2 (8.7)
Asian—no. (%)	0	1 (4.3)	1 (4.2)	1 (4.3)
Native American—no. (%)	0	0	0	1 (4.3)
Other race—no. (%)	1 (4.2)	0	1 (4.2)	0
Not Hispanic ethnic group—no. /group number (%)	23 (95.8)	22 (91.7)	23 (95.8)	22 (91.7)
Current smoker—no. (%)	4 (16.7)	2 (8.7)	2 (8.3)	2 (8.7)
Ex-Smoker—no. (%)	16 (66.7)	12 (52.2)	10 (41.7)	12 (52.2)
Never smoked—no. (%)	4 (16.7)	9 (39.1)	12 (50.0)	9 (39.1)
Currently consumes alcohol—no. (%)	20 (83.3)	16 (69.6)	18 (75.0)	20 (87.0)
Ex- Drinker of alcohol—no. (%)	2 (8.3)	5 (21.7)	4 (16.7)	3 (13.0)
Never consumes alcohol—no. (%)	2 (8.3)	2 (8.7)	2 (8.3)	0
Median body-mass index (IQR)	29.6 (28.0–34.0)	29.2 (26.4–32.6)	30.0 (27.1–32.5)	29.5 (27.7–32.9)
Hypertension—no. (%)	20 (83.3)	21 (91.3)	20 (83.3)	19 (82.6)
Myocardial infarction—no. (%)	10 (41.7)	12 (52.2)	12 (50.0)	14 (60.9)
History of percutaneous coronary intervention—no. (%)	20 (83.3)	15 (65.2)	17 (70.8)	19 (82.6)
History of coronary-artery bypass grafting—no. (%)	0	3 (13.0)	3 (12.5)	5 (21.7)
History of hyperlipidemia—no. (%)	22 (91.7)	23 (100.0)	21 (87.5)	23 (100.0)
History of congestive heart failure—no. (%)	1 (4.2)	0	0	1 (4.3)
Diabetes—no. (%)	8 (33.3)	11 (47.8)	12 (50.0)	8 (34.8)
Metabolic syndrome—no. (%)	23 (95.8)	22 (95.7)	22 (91.7)	22 (95.7)
Diabetes and metabolic syndrome—no. (%)	8 (33.3)	10 (43.5)	10 (41.7)	8 (34.8)
Use of ACE inhibitor or ARB—no. (%)	21 (87.5)	18 (78.3)	17 (70.8)	15 (65.2)
Use of statin—no. (%)	24 (100.0)	23 (100.0)	24 (100.0)	23 (100.0)
Use of beta-blocker—no. (%)	11 (45.8)	17 (73.9)	17 (70.8)	15 (65.2)
Use of antiplatelet or antithrombotic agent—no. (%)	14 (58.3)	9 (39.1)	13 (54.2)	9 (39.1)
Median high-sensitivity C-reactive protein level (IQR)	1.70 (0.80–3.03)	1.00 (0.80–2.20)	1.05 (0.60–2.48)	0.70 (0.40–1.15)

*One participant in the placebo arm self-identified with more than one race*.

After completing all screening procedures, qualifying subjects were randomly assigned to one of the following groups by the Johns Hopkins Investigational Pharmacy:

1) MTX: methotrexate (15 mg weekly) + placebo for colchicine (daily) + folate (1 mg daily);
2) LDC: colchicine (0.6 mg daily) + placebo for methotrexate (weekly) + folate (1 mg daily);
3) MTX+LDC: methotrexate (15 mg weekly) + colchicine (0.6 mg daily) + folate (1 mg daily); or
4) Placebo: placebo for methotrexate (weekly) + placebo for colchicine (daily) + folate (1 mg daily).

Folate was administered to reduce potential side effects of MTX and was given to all groups to avoid confounding. The investigators and participants were blinded to the study drug assignment. The study protocol was approved by the Institutional Review Board at The Johns Hopkins Hospital and University School of Medicine and complies with the Declaration of Helsinki. All participants provided written informed consent. Enrollment began April 2015 and the trial ended December 2018. Additional details appear in the Supplementary Material and the trial was registered at www.clinicaltrials.gov (NCT02366091).

### Study Procedures

Initial evaluation at baseline consisted of history, physical exam, and blood draw. Patients underwent MRI for CEF measures and brachial ultrasound for FMD at baseline, prior to study drug administration and after 8 and 24 weeks of study-drug administration. Study drug compliance was assessed by questionnaire and pill count at the 8, 16, and 24 week follow-up visits.

### MRI Methods for Coronary Endothelial Function (CEF)

Patients underwent MRI studies of CEF in the fasting state at baseline, 8- and 24-weeks using MRI methodology at rest and during continuous IHE as previously described (8, 15, 16). Detailed MRI parameters were previously published (15, 16), and further details are available in the Supplementary Material. Images were analyzed blinded to study-drug assignment and clinical information for CEF, as measured by change in cross-sectional area (CSA), coronary flow velocity (CFV), and coronary blood flow (CBF), as previously validated and described (15, 17). Our prior studies using this methodology demonstrated low intra- or inter-observer variability with good reproducility over 8 weeks (8).

### Systemic Endothelial Function and Inflammatory Biomarkers

Brachial flow mediated dilatation (FMD) and velocity were measured in the fasting state using standard techniques and analyzed in blinded fashion. Inflammatory biomarkers were measured at the University of Vermont (Supplementary Table 1).

### Sample Size

To test whether any of the anti-inflammatory strategies (i.e., LDC, MTX, and/or their combination) improves CEF in stable CAD patients with increased inflammation and abnormal CEF as compared to that of patients receiving placebo, this 2 × 2 factorial trial was designed with the primary endpoint of change in coronary cross-sectional area (CSA) from rest to that during IHE at 8 weeks. We chose this parameter because it reflects NO-dependent CEF and was shown to be reproducible over 8 weeks (8). CSA increases during IHE in healthy subjects and is unchanged or declines in patients with CAD (15, 18) and so the sample size was powered on the assumption that an anti-inflammatory medication would improve the CSA change to values midway between those of CAD patients and healthy subjects, in line with methotrexate-induced changes in FMD in patients (19) and the improvement in coronary endothelial function observed with statins (20). With a sample of 88 (22 in each cell), the power was 0.83 (alpha = 0.05, two-sided test) to detect a difference between the response in the placebo group and the response in each of the anti-inflammatory groups (8, 16). In November 2018, the CIRT trial was published showing no cardiovascular benefit of MTX (3). With guidance from the DSMB, this trial was stopped at a time when ~90% of the planned population had been enrolled (remaining power 0.8). Details of sample size calculations appear in the Supplementary Material.

### Statistical Approach

Demographic and baseline characteristics (e.g., age, race, sex, height, weight, etc.) were summarized using descriptive statistics for all participants. The primary analysis used an intent-to-treat approach. The primary efficacy endpoint was the % change in CSA from rest to IHE at the end of 8 weeks of the anti-inflammatory or placebo administration periods. The secondary efficacy endpoints included stress-induced change in CBF after 8 weeks of treatment, and change in CSA and CBF with IHE after 24 weeks of treatment. Further statistical details and methods are in the Supplementary Material.

## Results

There were no significant differences in baseline clinical and demographic characteristics among subjects randomized to the four study groups (Table 1). The median age was 63 years and 14% were women. Fifty-nine percent of the participants had metabolic syndrome with a median BMI of 29.5 kg/m^2^. Participants were clinically stable with a prior history of PCI in 75% and of prior myocardial infarction in 51%. Most patients were receiving guideline-recommended medical therapy for heart disease and all were on statin therapy. The median baseline hsCRP level for the entire cohort was 1.00 mg/L and the median low-density lipoprotein cholesterol level (LDL) was 74 mg/dL with no significant differences among the groups. Nine subjects qualified by hsCRP >2 mg/L criteria alone, 67 by diabetes/metabolic syndrome criteria, and 18 participants by both hsCRP and diabetes/metabolic syndrome. The disposition of subjects during the trial is shown in Figure 1.

### Primary and Secondary End Points, Coronary Endothelial Function

**Figure 2:**
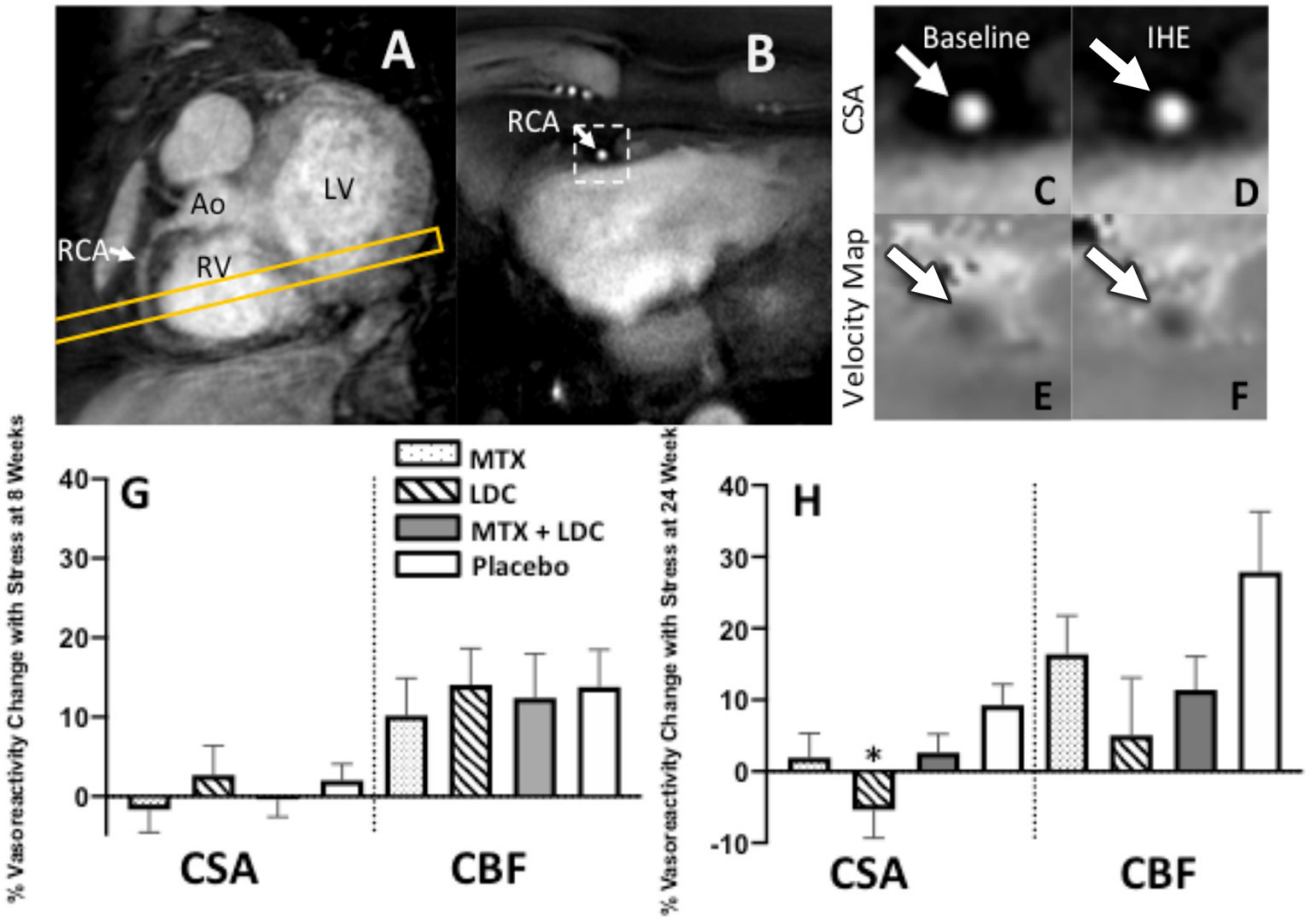
Representative coronary artery MRI images for CEF. **(A)** A scout MRI obtained parallel to the right coronary artery (RCA) is shown with the location for subsequent cross-sectional imaging (yellow outline). **(B)** Image acquired along the yellow-outlined region in **(A)** with RCA in cross-section (white arrow). The dotted rectangle in **(B)** is magnified in subsequent panels and shows the region analyzed for cross-sectional area at rest **(C)** and during exercise **(D)**. Flow velocity images of the same segment at rest **(E)** and during IHE **(F)** using a phase contrast technique wherein signal darkness increases only slightly during IHE, indicating an impaired velocity response. **(G,H)** Relative changes (%) in coronary artery cross sectional area (CSA), and coronary blood-flow (CBF) detected by MRI during isometric handgrip exercise at 8 weeks **(G)** and 24 weeks **(H)** for those on methotrexate (MTX, black), colchicine (gray), MTX and colchicine (red), and placebo (blue). Error bars indicate standard error of the mean. There were no significant differences in coronary endothelial function parameters between the placebo and anti-inflammatory treatments at the 8 week (primary) end point. % CSA change was lower in colchicine than placebo (*p = 0.02) at 24 weeks. Ao, aorta; LV, left ventricle; RV, right ventricle.

Representative images are shown in Figure 2. At baseline, the mean percent change in coronary cross sectional area (CSA) change with IHE for qualifying coronary segments was −11.8% ± 1.1% and for all coronary segments was +0.06% ± 1.1%. The percent change at baseline in the endpoint coronary blood flow (CBF) with IHE for qualifying coronary segments was +1.6% ± 3.9% and +14.3% ± 2.9% for all coronary segments, consistent with previously published studies in patients with CAD with endothelial dysfunction (15, 16).

The primary endpoint for the study, the change in CSA with IHE following 8 weeks of the anti-inflammatory treatments (MTX, LDC, or MTX + LDC) vs. placebo, did not differ among the study groups (Figure 2). Following 8 weeks of MTX, the mean IHE-induced percentage change in CSA for all segments was −1.7% ± 2.9%, following LDC: +2.7% ± 3.7%, following MTX+LDC: −0.4% ± 2.2%, and following 8 weeks of placebo was +2.0% ± 2.1% (*p* = NS, Figure 2G). Similarly, there were no significant differences in % CBF change with IHE at 8 weeks between groups (Figure 2G). Similarly, there were no differences among the groups in CEF at 8 weeks if only qualifying coronary segments were included in the analysis. In terms of other secondary endpoints such as CEF at 24 weeks, the % CSA change in the placebo arm was higher than that with colchicine (*p* = 0.02) but no significant differences in % CBF change among treatment groups were observed at 24 weeks (Figure 2H). Detailed CEF results are presented in Supplementary Tables 3, 4.

### Secondary Endpoints, Systemic Endothelial Function, and Inflammatory Markers

**Figure 3:**
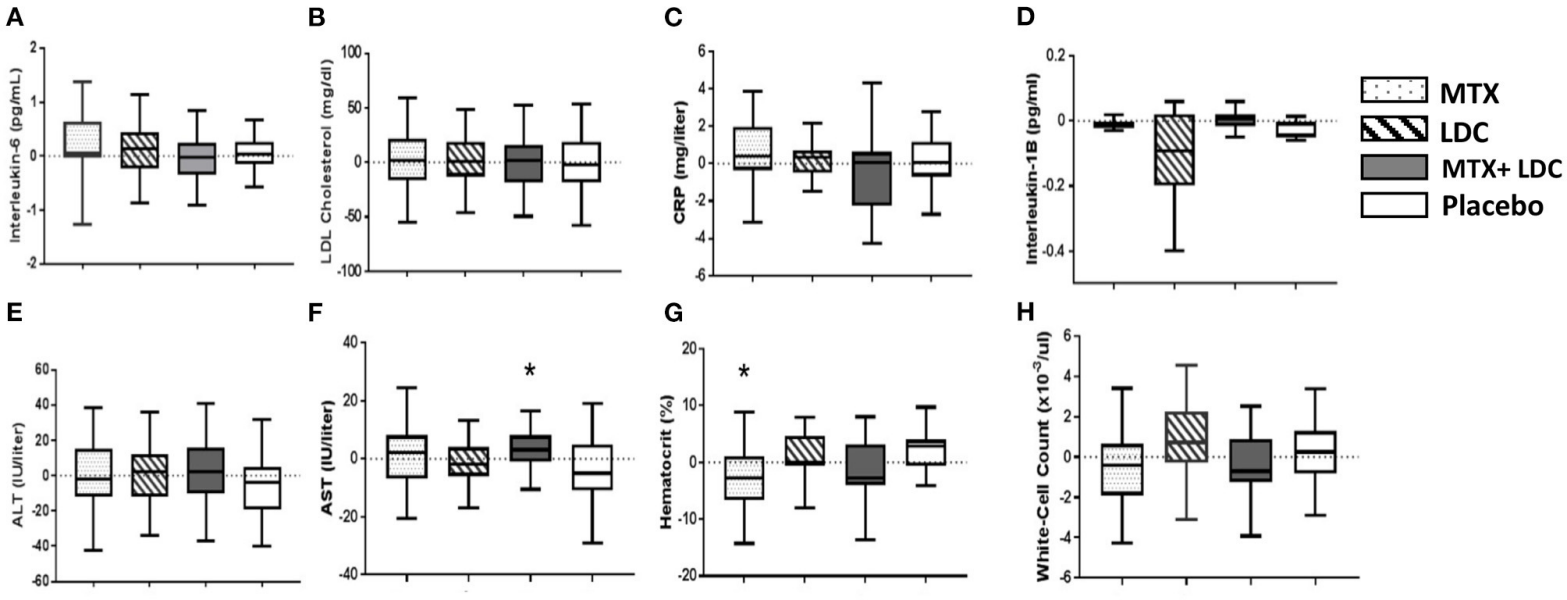
Bar graphs showing the effects of low-dose methotrexate (MTX, dotted bar), colchicine (LDC, striped bar), the combination of MTX and LDC (gray bar), and placebo (white bar) on **(A)**. interleukin-6, **(B)** low-density lipoprotein [LDL] cholesterol, **(C)** hsCRP, **(D)** interleukin-1β, hepatic enzyme levels **(E)** alanine aminotransferase [ALT] and **(F)** aspartate aminotransferase [AST], and hematologic measures **(G)** hematocrit level, **(H)** white-cell count. Data shown are the changes from study enrollment to 8 weeks after randomization. The horizontal line in each box represents the median, the top and bottom of the boxes represent the interquartile range, and the whiskers represent 1.5 times the interquartile range. *Statistically significant difference (*P* ≤ 0.05) compared to placebo.

At 8 weeks, administration of MTX, LDC, the combination or placebo did not result in significant changes in the inflammatory biomarkers of hsCRP, interleukin-6 (IL-6) or interleukin-1B from baseline values (Figure 3). At baseline, brachial FMD was 3.7% ± 0.3% (mean ± standard error) for all study participants with no significant differences among groups (Supplementary Table 3). There were also no differences in brachial FMD among groups after either 8 or 24 weeks of study drug administration (Supplementary Tables 3, 4).

### Safety

Overall, study treatment was relatively well-tolerated (Table 2). The most common adverse events were gastrointestinal disorders, minor infections and joint and muscle aches. There were very few serious AEs during the course of the study and no difference among study groups (Table 2). The reasons for premature withdrawal due to an AE are presented in Supplementary Table 2, with the most common reason being gastrointestinal complaints (3 in LDC group and 3 in MTX group). There were no significant changes in AST, ALT, white blood cell count, or LDL cholesterol at 8 weeks as compared to baseline. At 8 weeks, there were small but significant differences in AST level (between the placebo and MTX + LDC groups) and hematocrit (placebo vs. MTX) (Figure 3), as well as in thrombomodulin (placebo vs. colchicine) and sICAM3 (placebo vs. MTX + LDC) (Supplementary Table 3).

**Table 2:** Adverse events are shown in each study group.

**Adverse event or laboratory value (No. of patients)**	**Methotrexate (*n* = 24)**	**Colchicine (*n* = 23)**	**Methotrexate + Colchicine (*n* = 24)**	**Placebo (*n* = 23)**
Adverse events	41	38	46	33
Infection (respiratory or other)	8	8	9	6
Gastrointestinal disorder	9	9	7	2
Joint/Muscle soreness/Stiffness	5	2	7	7
Chest pain	1	1	1	1
Extremity swelling	1	2	0	1
Dental pain/Infection	0	1	2	1
Rash	2	2	1	0
Palpitations	1	1	1	0
Physical injury	4	2	1	2
Anxiety/Depression	0	1	3	0
Increased aspartate amino trans >3X the normal range	0	0	1	0
Increased alanine amino trans >3X the normal range	0	0	1	0
Decreased white blood cell count	1	2	1	3
Decreased hematocrit	4	6	8	6
Decreased Est GFR	5	1	3	4
Serious adverse event	3	2	0	1

*There were no significant differences in each adverse event among groups by Cox analysis*.

## Discussion

We believe this to be the first trial comparing different anti-inflammatory strategies in patients with CAD. In this randomized, double-blinded, placebo-controlled clinical trial, an anti-inflammatory approach with MTX, LDC or the combination of the two did not improve coronary endothelial dysfunction in stable CAD patients on statin therapy. Treatment with MTX, LDC, and the combination did not result in significantly more adverse events or serious adverse events compared to placebo (Table 2). Moreover, we observed that treatment with these anti-inflammatory agents did not result in reductions of serum markers of inflammation or improvements in systemic brachial endothelial function in patients with stable CAD.

Endothelial-dependent coronary vasoreactivity is an important index of vascular health and predicts cardiovascular events (6, 21). CEF is impaired early in the atherosclerotic process and can now be measured using novel non-invasive MRI methods (15). Recent studies demonstrate that MRI measures of CEF performed during IHE quantify nitric oxide-mediated coronary endothelial vasoreactivity with excellent short- and longer-term reproducibility (8, 15). Primary and secondary prevention medications such as statins and ACE-inhibitors improve CEF (7, 22). We measured endothelial function at 8 and 24 weeks because prior studies showed that statins rapidly improve endothelial function in the short term (days to weeks) (20, 23, 24) and in the longer term (5–6 months) (7). More recently, we observed that the PCSK9 inhibitor evolocumab improves CEF measured with these MRI techniques in just 6 weeks in patients with dyslipidemia and people living with HIV (9), indicating that the MRI-handgrip technique is sensitive enough to detect relatively rapid improvements in CEF in response to treatment more rapidly and in smaller cohorts than studied here.

A growing body of evidence suggests that inflammation plays an important role in coronary atherosclerosis and endothelial dysfunction, and there is heightened interest in using therapies that target inflammatory pathways to treat atherosclerosis and its complications (25). Recently, several large randomized clinical trials reported varying results using different anti-inflammatory approaches in CAD patients. The CANTOS (Canakinumab Anti-inflammatory Thrombosis Outcome Study) trial showed that the monoclonal antibody canakinumab directed against IL-1B was effective in reducing recurrent cardiovascular events in patients with prior MI and elevated CRP (4). Canakinumab reduced systemic biomarkers of inflammation and vascular events, but was associated with an increased risk of fatal sepsis compared to placebo (4). The more recent CIRT (Cardiovascular Inflammation Reduction Trial) study evaluated the effect of low dose MTX vs. placebo in CAD patients with metabolic syndrome or diabetes and residual increased inflammation, and found that MTX did not reduce inflammatory markers or events compared to placebo and was stopped prematurely due to futility (3). In the present study, we used a similar dose of MTX and many similar entry criteria and also observed that MTX did not lower markers of inflammation compared to placebo, and show here for the first time that MTX does not improve endothelial dysfunction at 8 and 24 weeks of administration.

Several recent trials evaluated the utility of colchicine to reduce cardiovascular events in patients with CAD (12, 26). While a higher dose of colchicine (0.5 mg twice daily) lowered CRP levels, a lower dose (0.5 mg daily) was used in subsequent endpoint trials (27). The LoDoCo-MI evaluated the acute effects of colchicine vs. placebo in patients following acute MI and with persistently elevated CRP (>2 mg/L), and found that colchicine did not reduce CRP levels 30 days after MI (28). The very recent and much larger COLCOT trial randomized acute MI patients to low does colchicine vs. placebo and reported a reduction in a composite endpoint of cardiovascular events driven by a lower incidence of stroke and hospitalization for angina in the colchicine group (12). However the inflammatory states in the setting of acute MI as studied in COLCOT compared to stable CAD as studied in the present trial are likely different (12). Moreover, CRP levels declined after MI on both study drugs in COLCOT and there was no difference in CRP decline between colchicine and placebo. Importantly, there was no significant difference in cardiac events (acute infarct and ACS) and the composite event difference was driven by a stroke benefit with colchicine compared to placebo. The most recent LoDoCo2 trial in patients with chronic CAD showed that colchicine reduced a composite primary end-point of cardiovascular death, spontaneous myocardial infarction, ischemic stroke, or ischemia-driven coronary revascularization events but increased the risk of death from non-cardiovascular causes (29). Inflammatory biomarkers were not reported in LoDoCo2 so it is unclear if the effects of colchicine on clinical endpoints were due to suppressing inflammation, duration of treatment or patient specific factors. However, a proteomic sub-study of LoDoCo2 reported that hsCRP and other inflammatory biomarkers were significantly reduced in the colchicine group after 30 days of treatment (30). Other studies have reported that colchicine favorably improves coronary plaque morphology (31) and may play a role in reducing local cardiac inflammatory cytokine production (32). Our study is the first to compare multiple anti-inflammatory medications in the same trial (LDC, MTX, LDC + MTX) and we observe that colchicine alone or in combination with methotrexate does not improve coronary endothelial dysfunction over the short and intermediate term in stable CAD patients on statins. The finding that our anti-inflammatory approach with MTX, LDC or the combination did not reduce inflammatory markers such as CRP is also consistent with the findings in other randomized clinical trials (CIRT, LODOCO-MI, and COLCOT) that reported a neutral effect of similar anti-inflammatory strategies on CRP and other inflammatory markers.

### Adverse Events

There were no significant differences in serious adverse events experienced during treatment with anti-inflammatory agents compared to placebo. All reported AEs were only mild-moderate in severity, and these findings confirm prior studies of the relative safety and tolerability of MTX and LDC. There are limited data using the combination of MTX and LDC which was not studied previously in CAD patients. A previous study in patients with primary biliary cirrhosis using the combination of LDC and MTX reported few side effects over a 3.4 year period (33). This is consistent with the results of our study, which provides important safety data for their combined use over a 24 week period in patients with stable CAD.

### Limitations

Our study was not powered for clinical outcomes but instead exploited powerful imaging approaches to directly evaluate coronary vascular health in response to the inflammatory interventions in a relatively modest sample size. The cohort size was justified with sample size estimates using prior published data of MRI measures of CE (8, 34). In addition, a prior study showed that this MRI-CEF approach can detect earlier improvements in CEF with PCSK9 inhibition in a smaller-sized cohort (9). The lack of trending differences for benefit among the study groups indicates that a considerably larger sample size is unlikely to have resulted in any significant group differences as well. It may be of interest in future trials to evaluate only subjects with biomarker evidence residual inflammatory risk, as done in the CANTOS trial (4). The MRI-CEF approach can safely detect coronary functional abnormalities in children and adolescents with type I diabetes (35) and thus can be applied to study vascular health and the impact of potential interventions across the lifespan.

### Conclusion

In summary, our study is the first to examine and compare the effects of anti-inflammatory approaches using MTX, LDC or the combination of the two on coronary endothelial dysfunction in patients with stable CAD and either elevated hsCRP or diabetes/metabolic syndrome on stable statin therapy. The anti-inflammatory agents MTX and LDC were generally well-tolerated; however, they did not improve coronary endothelial function, a well-established “barometer” of vascular health. Although MTX and LDC are commonly available, relatively inexpensive anti-inflammatory medications with well-known safety profiles, prior large trials suggest the benefits for cardiovascular disease are difficult to detect (MTX) or possibly limited to mostly cerebrovascular events in selected populations (colchicine). Although prior studies after CABG or post-MI suggested rapid effects of some of these agents to reduce inflammatory biomarkers (28), the current study demonstrates that these agents at these dosages do not reduce systemic markers of inflammation over 2 months in stable CAD patients. These findings suggest that the short-term and intermediate-term use of these anti-inflammatory approaches in stable CAD patients do not significantly improve either coronary artery or systemic endothelial function, both well-established predictors of cardiovascular outcomes and measures of vascular health.

## Data Availability Statement

The raw data supporting the conclusions of this article will be made available by the authors, without undue reservation.

## Ethics Statement

The studies involving human participants were reviewed and approved by Johns Hopkins Institutional Review Board. The patients/participants provided their written informed consent to participate in this study.

## Author Contributions

This randomized trial was designed by RW, AH, GG, and SL. Patient visits, blood sample collection and preparation, and/or data entry and quality assurance were performed by AS and SS. The MRI and ultrasound data were collected and/or analyzed by AH, MS, GB, YA, and JM. Statistical analysis was performed by SL. This manuscript was drafted by AH. Critically edited by AH, GG, SL, and RW. Funding was obtained by RW. All authors contributed to the article and approved the submitted version.

## Funding

This work was supported by the National Institutes of Health (HL120905 to RW, HL147660 to AH), the American Heart Association (AHA 17GRNT33670943 to AH), and the Clarence Doodeman Endowment in Cardiology at Johns Hopkins University School of Medicine.

## Conflict of Interest

The authors declare that the research was conducted in the absence of any commercial or financial relationships that could be construed as a potential conflict of interest.

## Publisher's Note

All claims expressed in this article are solely those of the authors and do not necessarily represent those of their affiliated organizations, or those of the publisher, the editors and the reviewers. Any product that may be evaluated in this article, or claim that may be made by its manufacturer, is not guaranteed or endorsed by the publisher.

## Abbreviations

CAD: coronary artery disease
CEF: coronary endothelial function
CSA: cross-sectional area
CBV: coronary blood velocity
CBF: coronary blood flow
FMD: flow mediated dilatation
LDC: low dose colchicine
MRI: magnetic resonance imaging
MTX: methotrexate
NO: nitric oxide
PCSK9: proprotein convertase subtilisin/kexin type 9.
