# Supplementary material for: Randomized Trial of Anti-inflammatory Medications and Coronary Endothelial Dysfunction in Patients With Stable Coronary Disease
Source: Front Cardiovasc Med. 2021 Oct 15;8:728654. doi: 10.3389/fcvm.2021.728654 (PMC8553961; doi:10.3389/fcvm.2021.728654)
Supplement: Supplementary file 1 [file Data_Sheet_1.pdf]

Supplement of detailed study procedures and endpoints:

Trial Objective:

The main objective of the trial was to determine, in patients with stable coronary artery disease and impaired coronary endothelial function (CEF), whether CEF is better in subjects receiving one of three anti-inflammatory regimens over eight to twenty four weeks than that of those who received placebo.

Detailed Study Procedures:

Entry Criteria

The inclusion criteria were 1) participants of either gender who were ≥21 years of age (no upper age limit), 2) history of prior MI, coronary revascularization, or coronary angiography or MDCT demonstrating at least one coronary artery with ≥50% luminal stenosis and no plans for revascularization, 3) clinically stable for 3 months, 3) vascular inflammation based on elevated hsCRP (>2mg L^{-1}) 4) abnormal CEF (change in CSA during IHE of ≤0% of the resting value: by this we mean any decrease in CSA or no change (0%) from baseline during IHE), 5) current treatment with a statin. During initial screening we noted that relatively few (<20%) of potential participants met the hsCRP≥2ml L^{-1} criterion, so after discussion with investigators, DSMB and NIH program officer, the criteria were changed to better align with the CIRT trial and thus after Feb 9, 2016, the third entry criterion was vascular inflammation based on elevated hsCRP (>2mg L^{-1}), or a clinical diagnosis of diabetes mellitus or metabolic syndrome, considered inflammatory syndromes. Metabolic syndrome is defined by three or more of the following:

1. Abdominal obesity (waist circumference: Men>102 cm (>40 in), Women >88 cm (>35 in)),
2. Serum triglycerides ≥150 mg/dL (or taking medication to treat high triglycerides),

3. HDL cholesterol: Men<40 mg/dL, Women<50 mg/dL (or taking medication to treat low HDL cholesterol),
4. High blood pressure: ≥130/≥85 mm Hg (or taking medication to treat high blood pressure), or
5. Fasting glucose: ≥100 mg/dL (or taking medication to treat high fasting glucose).

Subjects were excluded who: 1) were unable to understand the risks, benefits, and alternatives of participation and give meaningful consent, 2) had contraindications to MRI such as implanted metallic objects (pre-existing cardiac pacemakers, cerebral clips) or indwelling metallic projectiles, 3) had an acute coronary syndrome within the prior three months, 4) pregnant women, 5) had contraindications to methotrexate or colchicine as outlined by the American College of Rheumatology; including active bacterial infection, tuberculosis, or herpes zoster infection, leukopenia (<4000/mm^3), thrombocytopenia (<135,000/mm^3), elevation in hepatic transaminases (>2x upper limit of normal), hepatitis B or C, moderate renal disease (estimated creatine clearance <45ml/min), or planned surgery, 6) chronic inflammatory condition such as lupus or rheumatoid arthritis, ulcerative colitis or Crohn's disease, 7) Interstitial lung disease or pulmonary fibrosis, 8) were HIV positive, 9) had a requirement for, or intolerance to, methotrexate or colchicine, 10) intolerance to methotrexate, colchicine or folate, 11) history of non-basal cell malignancy or treatment for lymphoproliferative disease in the past 5 years, 12) requirement for use of drugs that alter folate metabolism, 13) history of alcohol abuse or unwillingness to limit consumption to < 4 drinks per week, 14) women of childbearing potential or intention to breastfeed, 15) men who plan to father children during the study period; men who have sexual intercourse with women of childbearing potential must agree to use a condom, 16) chronic use of oral or IV steroid therapy or other immunosuppressive or biologic response modifiers, 17) history of chronic pericardial effusion, pleural effusion or ascites, 18) New York Heart Association Class IV heart failure.

Initial evaluation: A careful history including whether angina was present and its severity by the Canadian Cardiovascular Society Angina Classification was obtained, and a physical examination performed. Blood samples were acquired for complete blood cell count, routine chemistry panel including hepatic transaminases and serum creatinine, LDL and HDL cholesterol and triglycerides, as well as serum hs-CRP, and other biomarkers (IL-6, IL-1β, IL-10, TNFα, IFN-γ, ICAM-1, sICAM-3, E-selectin, P-selectin, thrombomodulin, PCSK9). Patients were also tested for hepatitis B and C.

Follow up End-point Evaluation: At 8 and 24 weeks of study-drug administration, participants underwent repeat clinical evaluation, lipid and inflammatory biomarker analysis, as well as brachial ultrasound FMD and MRI for CEF with the same protocols used at baseline, prior to study drug administration. Study drug compliance was assessed by questionnaire and pill count at the 8, 16 and 24 week follow-up visits. To be compliant the participants had to be within 80%-100% compliance with taking the study medications. No participant was removed from the study for noncompliance.

Specific Methodologic Protocols:

MRI methods for Coronary Vasoreactivity: an index of CEF: CMR was performed on a 3 T CMR scanner (Achieva, Philips Healthcare, Best, NL) in the morning after an overnight fast. For endothelial function imaging, alternating anatomical and velocity-encoded images were collected at baseline and during approximately 6 min of continuous isometric handgrip exercise (IHE) as previously described.[1, 2] Images were taken perpendicular to a proximal or mid-straight segment of the coronary artery best identified on survey scans.[2] In particular, coronary MRI was repeated with an identical protocol with special attention taken to interrogate the same coronary segments

in follow-up at 8- and 24- weeks as those studied at baseline, using anatomic landmarks of coronary ostia and branch vessels- as we have done in the past[1] and to replicate the identical MRI IHE protocol. Cross-sectional anatomical (bright blood sequence) and phase contrast velocity encoded spiral CMR were obtained using single breath-hold cine sequences with reproducibility of the techniques published previously.[1, 3] The MRI was used to measure cross-sectional area (CSA), coronary flow velocity (CFV), and to calculate coronary blood flow (CBF) changes in response to IHE stress (continuous isometric handgrip for ~6 min at 30% of each subject's maximum force which was determined prior to entering the MRI), and as previously reported.[1, 3] Patients who qualified, based on the baseline MRI and other entry criteria, were randomized to one of the four study drug regimens and underwent repeat MRI at 8 and 24 weeks.

MRI analysis: Images were analyzed blinded to study-drug assignment and clinical information for CEF (e.g. change in cross-sectional area (CSA), coronary flow velocity (CFV), and coronary blood flow (CBF), as previously validated and described.[1, 3] Coronary CSA was quantified using a semi-automated software tool (Cine version 3.15.17, General Electric, Milwaukee, WI, USA) as previously reported[2] For CBV and CBF measurements, phase-contrast i*mages* were analyzed using semi-automated commercial software (FLOW Version 3.0, Medis, NL); the endpoint coronary artery blood-flow (CBF, in ml/minute) derived from diastolic coronary flow velocity (CFV, in cm/sec) as previously described.[1, 2] Only native coronary segments were analyzed and segments previously revascularized were excluded. In subjects in whom adequate image quality permitted the acquisition of CEF in multiple coronary artery segments, the results were averaged for all coronary segments for a given patient so that the results for patients with multiple acquisitions were not more heavily weighted than for those with fewer acquisitions. Our prior

studies using this methodology demonstrated low intra- or inter-observer variability with good reproducicility over eight weeks.[1]

Systemic Endothelial Function: Brachial Flow Mediated Dilatation (FMD) and Velocity: These studies were conducted in the fasting state (>10 hours) and participants were asked to refrain from drinking alcohol or beverages containing caffeine in the prior 24 h using a standardized protocol[4]. The brachial artery was visualized with a high-resolution 12 MHz ultrasound probe proximal to the antecubital fossa. After baseline images and flow measurements, a pressure cuff on the lower arm was inflated to 200–250 mmHg for 5 min. Blood flow and vessel diameter were continuously measured for approximately 2 minutes after cuff release (to identify the maximal vessel diameter and velocity). Flow-mediated dilation (FMD) and velocity time interval (VTI) were recorded and images were analyzed in blinded fashion using Vascular Research Tools 6 (Medical Imaging Applications, Coralville, IA, USA).

Blood Draw and Biomarker Analysis: Blood samples were obtained from a peripheral vein using standard venipuncture techniques. Blood specimens were collected in serum separator tubes and sodium citrate tubes.

The specimens in the sodium citrate tubes were centrifuged immediately after collection using a centrifuge with an integrated refrigeration system (at 4°C/1000 g for 10 min at 2300rpm) to separate plasma from blood cells. Following separation of plasma, approximately 3mL of plasma was extracted from the sodium citrate tubes into a labeled 15mL tube free of any anticoagulant agents. The labeled 15mL tube containing the plasma was then centrifuged again at 4°C/1000 g for 10 min at 4000rpm. The plasma was then divided into 1mL labeled cryovials and kept at -80°C. The specimens in the serum separator tubes were centrifuged 20 minutes after collection (time allowed for clotting) using a centrifuge with an integrated refrigeration system (at

4°C/1000 g for 20 min at 3000rpm). The serum was then divided into 1mL labeled cryovials and kept at -80°C.

Inflammatory biomarker analysis was performed by the Laboratory for Clinical Biochemistry Research at the University of Vermont using the Methods detailed in Table 1 below.

Supplement Table 1:

								Inter Assay CV for Controls		
Assay Performed	Manufacturer	Cat#	Method	Sample Type	Min Vol (uLs)	Dilution	Approx. Detectable Range	Serum Pool 1	EDTA Pool	Citrate Pool
hs-CRP	Siemens	10446091	Nephelometry	Serum	40-60	20	0.15-48.25 ug/mL	4.37	2.63	
IL-1β	Meso Scale	Pro-Inflammatory Panel	Electrochemiluminescense	Serum	80	2	0.067-1108 pg/mL	9.76	89.03	35.41
IL-6	Meso Scale	Pro-Inflammatory Panel	Electrochemiluminescense	Serum		2	0.091-1484 pg/mL	12.47	13.02	14.21
TNFa	Meso Scale	Pro-Inflammatory Panel	Electrochemiluminescense	Serum		2	0.042-698 pg/mL	8.85	8.72	7.19
IFNg	Meso Scale	Pro-Inflammatory Panel	Electrochemiluminescense	Serum		2	0.156-2560 pg/mL	11.18	10.27	9.78
IL-10	Meso Scale	Pro-Inflammatory Panel	Electrochemiluminescense	Serum		2	0.046-756 pg/mL	10.62	28.97	14.64
ICAM-1	R&D	DCD540	ELISA	Serum	40	20	31.2-1000 ng/mL	3.19	4.84	3.93
sE-selectin	Meso Scale	Human Vascular Injury Panel	Electrochemiluminescense	Serum	80	None	0.06-100 ng/mL	13.11	15.36	11.00
sP-selectin	Meso Scale	Human Vascular Injury Panel	Electrochemiluminescense	Serum		None	0.06-100 ng/mL	14.05	12.13	12.33
Thrombomodulin	Meso Scale	Human Vascular Injury Panel	Electrochemiluminescense	Serum		None	0.06-100 ng/mL	15.37	15.87	12.97
PCSK-9	R&D	DPC900	ELISA	Serum	40	20	12.5-*500* ng/mL	2.90	5.04	4.40

Details of laboratory testing for inflammatory markers. IL=interleukin, IFN=interferon, TNF alpha=tumor necrosis factor alpha, ICAM=intercellular adhesion molecule, PCKS9=proprotein convertase subtilisin/kexin type 9.

Trial Endpoints:

Primary Endpoint:

Coronary endothelial function at 8 weeks; specifically, change in coronary cross sectional area (CSA) from rest to that during isometric handgrip exercise (IHE) (with the change expressed as % of the resting value and as mm^2).

Secondary Endpoints

1. Change in coronary artery CSA from rest to IHE stress (as mm^2 and as % rest) at 24 weeks
2. Change in coronary blood velocity (CBV) from rest to IHE stress (as cm/s and as % rest) at 8 and 24 weeks
3. Change in coronary blood flow (CBF) from rest to IHE stress (as ml/min and as % rest) at 8 and 24 weeks
4. Difference between baseline and 8 and 24 weeks in IHE-induced changes in coronary CSA
5. Difference between baseline and 8 and 24 weeks in IHE-induced changes in coronary CBF

6. Difference between baseline and 8 and 24 weeks in IHE-induced changes in coronary CBV.

7. Serum hs-CRP at 8 weeks and change in hsCRP between baseline and 8 weeks.

8. Serum IL-6 at 8 weeks and change in IL-6 between baseline and 8 weeks.

9. Serum TNFα at 8 weeks and change in TNFα between baseline and 8 weeks.

10. Brachial flow mediated dilation (FMD) at 8 and 24 weeks and change in brachial FMD between baseline and 8 and 24 weeks.

11. The relationship between change in inflammatory markers (hsCRP, IL-6, TNFα, and others) between baseline and 8 weeks and change in CEF (IHE-induced change in CSA, CBV, CBF) between baseline and 8 weeks.

12. Safety endpoints (withdrawal due to side-effects, complete metabolic panel, including liver function tests, and complete blood count.)

Sample size calculation:

To answer the question of whether any of the anti-inflammatory strategies (i.e. LDC, MTX, and/or their combination) improves CEF in stable CAD patients with increased inflammation and abnormal CEF as compared to that of patients receiving placebo, this 2x2 factorial trial was designed with the primary endpoint of change in coronary cross-sectional area (CSA) from rest to that during IHE at 8 weeks. We chose this parameter because it reflects macrovascular coronary changes related to the endothelial-dependent stressor and because this parameter was shown to be reproducible over at least 8 weeks[1, 5]. CSA increases during IHE by 18%±13% (mean±SD) in healthy subjects and declines by -6%±11% (mean±SD) in patients with coronary atherosclerosis[1, 2]. We believe that a mean IHE-induced increase in CSA to +6% with IHE in the LDC or MTX groups would be physiologically significant. As the normal mean CSA response with IHE in healthy individuals is +18% and that in those with coronary atherosclerosis is -6%, then a mean change from -6% in placebo to +6% in the LDC or VLDM groups represents an improvement of

one-half of the difference between CAD and normal (i.e. 12 is one-half of the difference between the response in healthy individuals and those with CAD or -6 to +18= 24 units). Such a difference is in line with methotrexate-induced changes in FMD in RA patients RA patients[6, 7]. We will also measured CBF and CFV which have microvascular components and an endothelial-dependent component[2, 8].

Therefore, we assumed that at 8 weeks:

(1) Mean CSA would decrease by 6% from rest to IHE in CAD patients on placebo (**Δ** = -6%)

(2) Mean CSA would increase by +6% from rest to IHE in CAD patients on LDC (**Δ** = +6%)

(3) Mean CSA would increase by +6%from rest to IHE in CAD patients on MTX (**Δ** = +6%)

(4) Mean CSA would increase by +12% from rest to IHE in CAD patients on LDC+MTX (**Δ** = +12%)

(5) The pooled standard deviation for all four groups would be 11%.

With a sample of 88 (22 in each cell), the power would be 0.83 (alpha=0.05, two-sided test) to detect a difference between the response in the placebo group and the response in each of the three groups receiving the anti-inflammation intervention (LDC, MTX, LDC+MTX).[1, 3, 9] We believed this was a conservative estimate as it does not assume that a medication will improve CEF to normal values but instead will increase it by about 50% of the difference between healthy and CAD individuals, a physiologically relevant difference in line with changes observed in other settings.[6, 7, 10] Thus we anticipated enrolling enough participants to have 100-104 people who met entry criteria for randomization and, following drop-out, to ultimately have 22 in each of the 4 groups complete the eight week follow-up MRI examinations.

Statistical approach: Demographic and baseline characteristics (e.g. age, race, sex, height, weight, etc.) were summarized using descriptive statistics for all participants. Unless otherwise specified,

descriptive statistics on continuous variables consisted of the number of participants, and mean and standard deviation, or median and interquartile range.

The primary analysis used an intent-to-treat approach. The primary efficacy endpoint was the % change in CSA from rest to IHE at the end of 8 weeks of the anti-inflammatory or placebo administration periods. The secondary efficacy endpoints were stress-induced change in CBF after 8 weeks of treatment, and change in CSA and CBF with IHE after 24 weeks of treatment. The secondary endpoints also included brachial FMD and degree of change of inflammatory markers, after 8 weeks. There were no changes to trial outcomes after the study commenced.

One way of analysis of variance (ANOVA) with Tukey's pairwise comparisons was used to determine whether there were any statistically significant differences in change in CSA from rest to IHE stress at 8 weeks, between the 3 treatment groups (LDC, MTX, or MTX+LDL) with the placebo group. A generalized estimating equation (GEE) model was used to examine whether CEF changes between the baseline and 8 week studies were associated with treatment type (group assignment). A similar ANOVA approach was also repeated for 24-week data with Tukey's correction for multiple comparisons at each time point. To answer the questions of whether changes between baseline, 8 and 24 weeks in other indices of CEF (such as CBF) or in inflammatory biomarkers (hsCRP, IL-6, TNFα, etc.) differed between the groups (placebo vs LDC, placebo vs MTX, placebo vs MTX+LDC, a GEE approach was used. The p-value, least square (LS) treatment means, difference between the LS treatment means, and 95% confidence intervals for the treatment differences were determined. A similar approach was used to compare brachial ultrasound FMD among groups.

To determine whether there was a difference among groups in the proportion of participants who experienced abnormal laboratory values or withdrawal from the study, Chi-square analysis was performed. Finally, to determine whether there were differences in CEF or inflammatory

biomarkers among the treatment groups, the endpoints at 8 weeks were compared using ANOVA and the changes from baseline to 8 and to 24 weeks were compared with GEE. Unless otherwise specified, all statistical tests and confidence intervals were two-sided and conducted at the 0.05 significance level. Summary data are shown as mean and standard error or as median and interquartile range, when appropriate.

Overall, safety and tolerability were assessed by comparing the incidence of treatment-emergent adverse events, laboratory tests, vital signs, and other safety variables among groups. Adverse events were summarized based on the study drug assignment at the time of the event using the full analysis data set. Laboratory test variables were summarized by study drug and visit using descriptive statistics.

Safety surveillance: Participants underwent surveillance safety monitoring every 4 weeks and included complete blood counts, liver function tests, and BUN and creatinine levels. Abnormal results were reviewed for safety with the Data and Safety Monitoring Board Chair on a regular basis. Participants were asked to return 4 weeks after the last dose of study drug (i.e. 28 weeks after randomization and initial dosing) for a safety evaluation.

If any of the following criteria were met (CBC<3000, platelets<50,000, Hct<27%, GFR <30ml/min or a >50% reduction in GFR compared to baseline values, and LFTs >3x upper limit of normal), the laboratory test was repeated. If confirmed on repeat, there was a temporary discontinuation of the study drugs. If the abnormality resolved, the study drugs were resumed at the same or lower dose. If the abnormality did not resolve, then the case was reviewed for discontinuation from the study. No participant had study drug permanently discontinued because of laboratory abnormalities.

Reasons for withdrawal from the trial are presented in Supplement Table 2. Serious adverse events (SAEs) were those that resulted in death, were life- threatening, led to hospitalization, caused clinically significant incapacity, or were deemed to be an important medical event as determined by the investigator. SAEs were as follows: Hospitalization for unstable angina and hospitalization for pulmonary symptoms (colchicine); Hospitalization for shoulder pain, hospitalization for acute shoulder pain, hospitalization for transient bilateral vision loss (methotrexate); Hospitalization for chest pain (placebo). There were no significant differences in AEs or SAE's among groups.

Supplement Table 2: Reasons for participant withdrawal

Reason for Withdrawal	MTX + Colchicine (n=2)	Colchicine (+ placebo for MTX) (n=9)	MTX (+ placebo for Colchicine) (n=7)	Placebo for both MTX and Colchicine (n=1)
**GI Complaints**		3	3	
**Claustrophobia**		1		
**Depression**	1			
**Subjective hair loss**		1		
**Joint stiffness**			1	
**New cancer diagnosis**	1			
**Suspected cancer**		1		
**Underwent surgery**			1	
**Chronic URI symptoms**		1		
**Started new medication**			1	
**ETOH restriction**		1		
**Time commitment**			1	1
**Personal reasons**		1		

Clinical Trial Conclusion:

On November 10, 2018, the results of the Cardiovascular Inflammation Reduction Trial (CIRT) were presented at the American Heart Association Meeting and published in *The New England Journal of Medicine.[11]* This double-blind randomized trial of low dose methotrexate vs placebo in 4786 patients with coronary artery disease and either diabetes or metabolic syndrome was stopped by the DSMB after a median follow up of 2.3 years and showed no significant differences between the two groups on the primary composite endpoint (nonfatal MI, nonfatal stroke and cardiovascular death) or on other predefined outcomes. Methotrexate was associated with elevations in liver-enzyme levels, reductions in leukocyte counts and hematocrit levels, and a higher incidence of non–basal-cell skin cancers than was placebo.

The CIRT findings are relevant for this Inflammation and Coronary Endothelial Function (InCEF) trial because CIRT studied a similar patient population, used nearly identical methotrexate dosing, and was designed to determine a mechanism responsible for the hypothesized cardiovascular benefit of an anti-inflammatory effect achieved by methotrexate and/or colchicine. The CIRT findings that low dose methotrexate in this population did not reduce serum inflammatory markers or events suggested strongly that low dose methotrexate was unlikely to improve coronary endothelial function in InCEF and unlikely to be used in clinical practice to decrease the likelihood of atherosclerotic events. Given this new information, it became difficult to justify the continued inconvenience and potential risks to participants as well as the cost to continue the low dose methotrexate arms in InCEF.

Because 80 of a targeted 88 participants had reached the primary endpoint (8 week MRI, or ~90% of that projected population had been enrolled in early November 2018, and because InCEF had a factorial design that would be compromised by stopping the two methotrexate arms of the four total arms, we performed updated sample size calculations to determine whether InCEF would be adequately powered if enrollment stopped then. Using the same assumptions in the

original sample size calculations, we calculated that if InCEF was terminated with 80 participants with primary endpoint data, the power to detect a significant difference between the placebo group and any of the intervention groups (colchicine, methotrexate, or colchicine/methotrexate) was ≥80%. Taking into consideration all of this information, the investigators terminated enrollment in the InCEF trial after discussion and approval by the DSMB and the NIH program officer. Final MRI and laboratory studies were obtained in those nearing an endpoint.

Follow up results for brachial FMD: Baseline and 8 week FMD results are presented in Supplement Table 3. At 24 weeks, there was no significant change in FMD compared to baseline in any 4 treatments groups and no differences among groups (Supplement Table 4).

Supplement Table 3:

		Baseline					8 Weeks				
	**Variable**	**Mean**	**Std Error**	**Median**	**Lower Quartile**	**Upper Quartile**	**Mean**	**Std Error**	**Median**	**Lower Quartile**	**Upper Quartile**
	% CSA with stress	2.30	2.68	0.53	-2.94	9.11	-1.70	2.85	-0.56	-7.94	5.58
	%CBF with stress	27.32	7.37	19.99	5.22	45.59	10.23	4.70	2.74	-1.09	14.51
	% Qualifying CSA with stress	-10.33	1.97	-8.44	-14.74	-1.39	-1.12	2.34	-0.01	-7.99	5.75
	% Qualifying CBF with stress	13.26	8.56	3.79	-14.17	33.21	14.51	6.76	12.74	-6.65	24.35
	Brachial FMD (%)	3.96	0.61	3.74	1.59	5.61	4.37	0.70	3.21	1.60	7.19
	FMD Systolic Shear Rare	233.22	13.49	219.16	184.73	263.48	252.05	14.75	234.32	222.60	283.75
	C reactive Protein	2.51	0.74	1.22	0.61	3.04	2.40	0.54	1.43	0.79	3.11
	IL-6	1.25	0.36	0.85	0.48	1.18	1.61	0.55	0.88	0.54	1.56
MTX (n=24)	TNF alpha	3.40	0.12	3.39	2.97	3.85	3.45	0.14	3.57	3.12	3.73
	IL Beta mean	0.07	0.01	0.05	0.04	0.08	0.09	0.03	0.06	0.04	0.07
	IL-10	0.32	0.03	0.28	0.23	0.38	0.32	0.02	0.30	0.24	0.34
	IFN-gamma	3.03	0.36	2.58	1.94	3.53	6.78	2.45	2.94	1.88	3.70
	E-selectin	16.78	1.67	14.35	11.64	18.66	16.92	1.64	16.12	11.08	19.58
	P-selectin	74.56	3.35	74.76	58.91	85.07	68.71	3.59	69.01	54.03	82.25
	Thrombomodulin	3.57	0.18	3.54	3.15	3.85	3.73	0.23	3.78	3.06	4.21
	ICAM1	222.55	14.44	217.18	172.15	249.94	207.03	12.27	206.12	186.59	231.80
	sICAM-3	1.51	0.06	1.52	1.36	1.68	1.43	0.09	1.38	1.16	1.62
	PCSK9	354.12	12.93	368.31	307.21	386.88	358.04	14.84	342.60	312.22	430.90
	% CSA with stress	-0.27	2.56	-0.46	-7.87	6.78	2.71	3.74	-1.47	-4.21	6.37
	%CBF with stress	6.60	6.17	3.60	-12.45	22.37	14.06	4.57	13.73	2.42	26.38
	% Qualifying CSA with stress	-9.98	1.46	-8.24	-14.95	-4.70	0.39	3.77	-1.11	-6.04	5.40
	% Qualifying CBF with stress	-4.07	6.83	-4.07	-22.69	16.89	10.72	7.23	11.65	-9.79	20.31
	Brachial FMD (%)	3.27	0.27	3.18	2.27	3.93	3.23	0.52	3.73	1.70	4.43
	FMD Systolic Shear Rare	229.91	16.96	202.78	176.23	262.22	225.80	15.89	209.16	183.67	245.05
	C reactive Protein	2.35	0.66	1.22	0.80	2.79	3.10	1.68	0.96	0.67	2.10
	IL-6	1.08	0.17	0.73	0.50	1.32	1.20	0.27	0.83	0.61	1.38
Colchicine (n=23)	TNF alpha	6.66	3.11	3.32	2.80	3.97	3.25	0.15	3.02	2.68	3.80
	IL Beta mean	0.05	0.01	0.04	0.03	0.06	0.05	0.00	0.04	0.03	0.06
	IL-10	0.37	0.05	0.31	0.23	0.41	0.35	0.05	0.27	0.23	0.36
	IFN-gamma	36.99	31.92	3.00	1.69	8.23	3.38	0.63	2.23	1.99	3.53
	E-selectin	14.67	1.68	15.69	9.68	19.35	13.64	1.46	11.66	9.97	18.75
	P-selectin	70.05	4.96	70.70	45.22	83.14	70.46	4.31	66.47	60.80	80.94
	Thrombomodulin	3.00	0.14	3.08	2.64	3.21	2.96	0.16	2.90	2.39	3.31
	ICAM1	204.77	13.69	206.77	169.41	232.11	210.50	9.96	199.67	182.07	221.51
	sICAM-3	1.61	0.20	1.53	1.10	1.71	1.34	0.11	1.30	1.02	1.47
	PCSK9	347.15	21.88	365.06	296.77	412.43	395.88	22.46	393.16	340.13	468.81
	% CSA with stress	-0.14	1.61	-0.68	-4.13	2.84	-0.39	2.23	-0.05	-6.50	4.99
	%CBF with stress	17.80	3.61	13.95	5.15	32.84	12.38	5.58	11.70	3.22	18.52
	% Qualifying CSA with stress	-13.53	2.61	-8.06	-22.04	-4.63	-2.79	3.35	-2.01	-12.32	4.82
	% Qualifying CBF with stress	4.58	9.38	3.98	-11.49	12.67	10.56	5.39	14.78	4.27	21.10
	Brachial FMD (%)	3.03	0.54	2.42	0.98	4.40	3.58	0.48	3.68	1.71	4.69
	FMD Systolic Shear Rare	223.12	10.29	212.31	182.90	262.11	203.69	13.03	209.94	172.18	246.45
	C reactive Protein	2.38	0.42	1.52	0.71	3.58	1.45	0.22	1.09	0.88	1.71
	IL-6	2.35	1.38	0.83	0.69	1.36	0.83	0.07	0.77	0.65	0.96
MTX+ colchicine (n=23)	TNF alpha	3.53	0.25	3.39	2.88	4.12	3.49	0.25	3.33	3.03	4.12
	IL Beta mean	0.04	0.00	0.04	0.03	0.05	0.05	0.01	0.05	0.04	0.07
	IL-10	0.31	0.03	0.28	0.20	0.36	0.33	0.04	0.31	0.19	0.39
	IFN-gamma	3.10	0.58	2.02	1.47	3.60	4.01	0.90	2.66	1.78	4.11
	E-selectin	17.71	1.26	16.21	12.30	22.67	16.18	1.24	15.24	11.36	20.30
	P-selectin	75.36	5.30	72.64	53.59	87.98	73.14	4.52	69.86	63.77	75.65
	Thrombomodulin	3.68	0.22	3.74	3.02	3.98	3.78	0.25	3.72	2.87	4.26
	ICAM1	225.51	12.70	228.51	175.89	263.55	209.81	10.59	205.73	190.63	233.19
	sICAM-3	1.36	0.09	1.22	1.07	1.49	1.27	0.06	1.22	1.06	1.39
	PCSK9	337.26	17.13	329.63	302.01	405.69	359.48	17.52	356.51	304.89	404.17
	% CSA with stress	-1.72	2.06	-2.12	-5.76	5.46	2.04	2.05	1.28	-5.13	10.74
	%CBF with stress	5.30	4.58	10.16	-9.95	19.42	13.81	4.68	8.17	-1.50	23.72
	% Qualifying CSA with stress	-13.36	2.20	-10.65	-23.64	-5.75	-2.59	3.25	-2.23	-7.61	8.41
	% Qualifying CBF with stress	-7.23	4.60	-10.63	-14.82	1.74	11.04	5.70	6.30	-9.23	27.92
	Brachial FMD (%)	4.05	0.54	3.59	2.73	4.74	4.57	0.60	4.34	2.44	5.88
	FMD Systolic Shear Rare	222.88	15.38	212.86	169.95	260.52	197.39	12.15	191.84	164.93	217.73
	C reactive Protein	1.30	0.30	0.88	0.45	1.23	1.65	0.39	0.98	0.46	1.75
	IL-6	1.05	0.20	0.77	0.65	1.11	0.92	0.07	0.90	0.70	1.19
Placebo (n=24)	TNF alpha	3.80	0.16	3.53	3.33	4.18	3.78	0.12	3.69	3.35	4.13
	IL Beta mean	0.06	0.01	0.04	0.03	0.05	0.07	0.02	0.04	0.03	0.06
	IL-10	0.41	0.07	0.30	0.26	0.47	0.39	0.05	0.34	0.26	0.41
	IFN-gamma	6.34	2.94	3.17	2.60	4.45	3.93	0.27	4.07	3.15	4.32
	E-selectin	14.81	1.38	14.47	8.80	18.59	15.57	1.53	14.59	10.77	18.50
	P-selectin	77.23	4.36	72.86	61.87	99.89	74.92	4.66	71.15	62.37	85.74
	Thrombomodulin	3.76	0.22	3.56	3.11	4.58	3.84	0.22	3.73	3.22	4.55
	ICAM1	230.09	14.38	211.61	181.90	261.44	230.00	11.79	213.98	191.95	252.62
	sICAM-3	1.58	0.10	1.45	1.22	1.85	1.62	0.10	1.47	1.32	1.88
	PCSK9	334.77	18.33	320.26	266.78	383.04	348.52	22.34	366.25	260.61	411.46

Results showing the primary endpoint (coronary endothelial function (CEF): % coronary cross sectional area (CSA) change with isometric handgrip handgrip stress) at baseline and after 8 weeks randomization to one of the four groups: methotrexate (MTX), colchicine, MTX with colchicine and placebo. Secondary endpoints including CEF endpoint coronary blood flow (CBF), and brachial flow mediated dilation (FMD), FMD systolic shear rate and serum inflammatory and endothelial markers are shown. IL=interleukin, IFN=interferon, TNF alpha=tumor necrosis factor alpha, ICAM=intercellular adhesion molecule, PCKS9=proprotein convertase subtilisin/kexin type 9. The units for CRP are mg/L; for E-selectin ng/mL, P-selectin ng/mL, Thromdomodulin ng/mL, ICAM1 ng/mL, sICAM3 ng/mL, PCSK9 ng/ml, and for IL-6, TNF alpha, IL-Beta mean, IL-10, IFN-gamma are pg/ml.

Supplement Table 4: Results after 24 weeks from randomization

	Variable	Mean	Std Dev	Std Error	Median	Lower Quartile	Upper Quartile
**MTX**	**% CSA with stress**	1.94	14.24	3.36	5.29	-7.44	12.67
	**% CBF with stress**	16.38	22.69	5.35	12.16	-1.10	33.83
	**% Qualifying CSA with stress**	-0.14	18.27	4.57	-2.15	-11.11	15.23
	**% Qualifying CBF with stress**	9.48	31.60	7.90	9.82	-5.34	38.29
	**Brachial FMD (%)**	4.69	3.00	0.73	4.29	3.43	5.95
**LDC**	**% CSA with stress**	-5.39	13.55	3.91	-3.50	-11.96	2.34
	**% CBF with stress**	5.05	26.77	8.07	7.46	-22.73	24.32
	**% Qualifying CSA with stress**	-10.64	14.31	4.31	-15.69	-18.30	0.36
	**% Qualifying CBF with stress**	0.91	20.38	6.44	4.53	-8.28	8.54
	**Brachial FMD (%)**	4.20	3.17	1.00	2.91	2.13	5.60
**MTX + LDC**	**% CSA with stress**	1.99	10.78	2.54	0.66	-5.47	7.65
	**% CBF with stress**	10.14	19.44	4.58	8.76	-4.79	28.11
	**% Qualifying CSA with stress**	5.24	20.94	4.93	-2.09	-6.06	13.99
	**% Qualifying CBF with stress**	14.88	20.22	4.90	12.56	-2.57	31.13
	**Brachial FMD (%)**	3.37	1.89	0.47	3.10	2.04	4.39
**Placebo**	**% CSA with stress**	9.26	13.02	2.91	6.95	1.49	17.02
	**% CBF with stress**	27.91	36.55	8.39	31.06	1.36	67.92
	**% Qualifying CSA with stress**	8.65	18.48	4.24	8.63	-3.84	21.53
	**% Qualifying CBF with stress**	27.41	46.05	11.17	17.07	0.32	22.93
	**Brachial FMD (%)**	3.55	2.78	0.61	3.82	1.33	5.29

Results showing secondary endpoints (coronary endothelial function (CEF): % coronary cross sectional area (CSA), % coronary blood flow (CBF) change with isometric handgrip stress, and systemic endothelial function with brachial flow mediated dilation (FMD) after 24 weeks randomization to one of the four groups: 1) methotrexate (MTX), 2) low dose colchicine (LDC), 3) MTX and LDC and 4) placebo.

References for Supplement

1. Hays AG, Iantorno M, Soleimanifard S, Steinberg A, Schar M, Gerstenblith G, et al. Coronary vasomotor responses to isometric handgrip exercise are primarily mediated by nitric oxide: a noninvasive MRI test of coronary endothelial function. Am J Physiol Heart Circ Physiol. 2015;308(11):H1343-50. Epub 2015/03/31. doi: 10.1152/ajpheart.00023.2015. PubMed PMID: 25820391; PubMed Central PMCID: PMCPMC4451304.
2. Hays AG, Hirsch GA, Kelle S, Gerstenblith G, Weiss RG, Stuber M. Noninvasive visualization of coronary artery endothelial function in healthy subjects and in patients with coronary artery disease. J Am Coll Cardiol. 2010;56(20):1657-65. doi: 10.1016/j.jacc.2010.06.036. PubMed PMID: 21050976.
3. Hays AG, Iantorno M, Schar M, Mukherjee M, Stuber M, Gerstenblith G, et al. Local coronary wall eccentricity and endothelial function are closely related in patients with atherosclerotic coronary artery disease. J Cardiovasc Magn Reson. 2017;19(1):51. Epub 2017/07/07. doi: 10.1186/s12968-017-0358-2. PubMed PMID: 28679397; PubMed Central PMCID: PMCPMC5499038.
4. Corretti MC, Anderson TJ, Benjamin EJ, Celermajer D, Charbonneau F, Creager MA, et al. Guidelines for the ultrasound assessment of endothelial-dependent flow-mediated vasodilation of the brachial artery: a report of the International Brachial Artery Reactivity Task Force. J Am Coll Cardiol. 2002;39(2):257-65. PubMed PMID: 11788217.
5. Hays AG, Stuber M, Hirsch GA, Yu J, Schar M, Weiss RG, et al. Non-invasive detection of coronary endothelial response to sequential handgrip exercise in coronary artery disease patients and healthy adults. PloS one. 2013;8(3):e58047. doi: 10.1371/journal.pone.0058047. PubMed PMID: 23536782; PubMed Central PMCID: PMC3594224.
6. Bergholm R, Leirisalo-Repo M, Vehkavaara S, Mäkimattila S, Taskinen MR, Yki-Järvinen H. Impaired responsiveness to NO in newly diagnosed patients with rheumatoid arthritis. Arteriosclerosis, Thrombosis, and Vascular Biology. 2002;22(10):1637-41.
7. Hjeltnes G, Hollan I, Førre Ø, Wiik A, Lyberg T, Mikkelsen K, et al. Endothelial function improves within 6 weeks of treatment with methotrexate or methotrexate in combination with a TNF-α inhibitor in rheumatoid arthritis patients. Scandinavian Journal of Rheumatology. 2012;41(3):240-2. doi: 10.3109/03009742.2012.656698.
8. Iantorno M, Hays AG, Schar M, Krishnaswamy R, Soleimanifard S, Steinberg A, et al. Simultaneous Noninvasive Assessment of Systemic and Coronary Endothelial Function. Circ Cardiovasc Imaging. 2016;9(3):e003954. Epub 2016/02/28. doi: 10.1161/CIRCIMAGING.115.003954. PubMed PMID: 26919997; PubMed Central PMCID: PMCPMC4839535.
9. Iantorno M, Soleimanifard S, Schar M, Brown TT, Bonanno G, Barditch-Crovo P, et al. Regional coronary endothelial dysfunction is related to the degree of local epicardial fat in people with HIV. Atherosclerosis. 2018;278:7-14. Epub 2018/09/19. doi: 10.1016/j.atherosclerosis.2018.08.002. PubMed PMID: 30227267; PubMed Central PMCID: PMCPMC7069555.
10. Hamilton SJ, Chew GT, Watts GF. Coenzyme Q10 improves endothelial dysfunction in statin-treated type 2 diabetic patients. Diabetes Care. 2009;32(5):810-2.

11. Ridker PM, Everett BM, Pradhan A, MacFadyen JG, Solomon DH, Zaharris E, et al. Low-Dose Methotrexate for the Prevention of Atherosclerotic Events. N Engl J Med. 2019;380(8):752-62. doi: 10.1056/NEJMoa1809798. PubMed PMID: 30415610; PubMed Central PMCID: PMCPMC6587584.
